# The smell of success: the amount of prey consumed by predators determines the strength and range of cascading non-consumptive effects

**DOI:** 10.7717/peerj.1426

**Published:** 2015-11-19

**Authors:** Marc Weissburg, Jeffrey Beauvais

**Affiliations:** School of Biology, Georgia Institute of Technology, Atlanta, GA, USA

**Keywords:** Chemical ecology, Non-consumptive effects, Risk sensitive behavior, Oysters, Blue crabs, Risk assessment, Trophic cascades

## Abstract

We examined whether chemically mediated risk perception by prey and the effects of changes in prey behavior on basal resources vary as a function of the amount of prey biomass consumed by the predator. We studied these issues using a tritrophic system composed of blue crabs, *Callinectes sapidus* (top predator), mud crabs *Panopeus herbstii* (intermediate prey), and oysters *Crassostrea virginica* (basal resource). Working in a well characterized field environment where experiments preserve natural patterns of water flow, we found that biomass consumed by a predator determines the range, intensity and nature of prey aversive responses. Predators that consume large amounts of prey flesh more strongly diminish consumption of basal resources by prey and exert effects over a larger range (in space and time) compared to predators that have eaten less. Less well-fed predators produce weaker effects, with the consequence that behaviorally mediated cascades preferentially occur in refuge habitats. Well-fed predators affected prey behavior and increased basal resources up to distances of 1–1.5 m, whereas predators fed restricted diet evoked changes in prey only when they were extremely close, typically 50 cm or less. Thus, consumptive and non-consumptive effects may be coupled; predators that have a greater degree of predatory success will affect prey traits more strongly and non-consumptive and consumptive effects may fluctuate in tandem, with some lag. Moreover, differences among predators in their degree of prey capture will create spatial and temporal variance in risk cue availability in the absence of underlying environmental effects.

## Introduction

Prey behavior, life-history and morphology all can be altered by cues from predators. These responses to predators, often called non-consumptive effects (NCEs), have strong impacts on prey demography and interactions with other organisms (e.g., Preisser, Bolnick & Benard, 2005). These responses also have energetic costs or can result in the allocation of time and energy away from other important activities, and so should, in theory, be calibrated to the degree of threat imposed by the predator (i.e., the likelihood this predator will consume the prey Chivers et al., 2001; Ferrari, Sih & Chivers, 2009). It is therefore essential to understand how prey species judge predator threat, and identify what properties of sensory cues available to prey allow them to estimate how much danger a predator represents. The need to examine how sensory cues convey threat is heightened by the fact that NCEs not only have direct effects on prey, but can cascade to other community members. Recent analysis suggests that these cascading effects (e.g., behaviorally-mediated trophic cascades) can be more important than the direct effects that occur when predators consume prey (Preisser, Bolnick & Benard, 2005).

Chemical cues from predators are ubiquitous and are important in generating NCEs on focal prey and in producing cascading effects (Chivers & Smith, 1998; Kats & Dill, 1998). In fact, the majority of studies on NCEs have examined systems where predator prey interactions are mediated by chemical cues (Weissburg, Smee & Ferner, 2014). Despite the hundreds of studies on chemically-mediated predator detection, we know little about how the degree of predator threat is conveyed by chemical cues. For instance, predator diet type has a strong effect on prey responses; in many (Chivers & Mirza, 2001; Schoeppner & Relyea, 2005; Turner, 2008), although not all (Smee & Weissburg, 2006) cases, prey respond more strongly to cues produced by predators that have eaten conspecifics relative to cues released by predators that have consumed different species. It is not clear why diet makes a difference in some but not all cases or what properties of cues released by predators are responsible for diet-dependent differences in prey response intensity. The amount of prey consumed by predators might be predicted to affect prey responses by modulating the amount, or concentration, of bioactive molecules released, which constitute potential cues associated with predation intensity; to our knowledge this effect never has been examined. Some studies deliberately have manipulated predator number, showing that prey responses increase when more predators are allowed to feed on prey (Chivers et al., 2001; Relyea, 2003; Hill & Weissburg, 2013b). This suggests altering the amount of prey flesh consumed by a predator also may mediate responses of focal prey and the consequences for other species (basal resources, competitors) that interact with this prey.

We examined the role of predator diet (amount of prey flesh consumed) in modulating threat sensitive behavior in a tritrophic system composed of blue crabs, *Callinectes sapidus* (top predator), mud crabs *Panopeus herbstii* (intermediate prey), and oysters *Crassostrea virginica* (basal resource). Mud crabs are small (adult size is 20–40 mm carapace width) cryptic xanthid crabs that live in association with intertidal and subtidal oyster reefs (Seed, 1980; Lee & Kneib, 1994). They are important and efficient consumers of juvenile oysters (<30 mm, referred to as oyster spat) as well as organisms that occupy interstices in the oyster reef matrix (Seed, 1980; Bisker & Castagna, 1987; Toscano & Griffen, 2012; Hill & Weissburg, 2013a). Numerous studies have demonstrated that oyster reefs provide mud crabs with a refuge from predation by a variety of invertebrate and vertebrate estuarine top predators, including toad fish (*Opsanus tau*) and blue crabs (Grabowski, 2004; Grabowski & Kimbro, 2005; Hill & Weissburg, 2013a). Blue crabs are an important predator of mud crabs; although blue crabs are generalist and opportunistic feeders, xanthid crabs compose roughly 10–40% of blue crab diets in the field (Laughlin, 1982; Fitz & Weigert, 1991) and appear to be a major predator of mud crabs in Wassaw sound (Hill & Weissburg, 2013a). The presence of blue crabs (Grabowski, 2004; Hill & Weissburg, 2013b) or water in which blue crabs were kept (Hill & Weissburg, 2013b) reduces activity and foraging in mud crabs, suggesting mud crab responses to blue crab predators are at least partially the result of chemical cues.

We examined predator properties that determine the strength of mud crab aversive behaviors in a semi-natural setting in the field. Our specific goals were to determine how blue crab diet (amount of mud crab prey consumed) affects the distance over which chemical signals affect mud crab prey, whether aversive responses of mud crabs were diet dependent, and how diet dependent responses of prey propagate to basal resources.

## Methods

### Animal collection and care

Mud crabs and blue crabs were collected locally from Wassaw Sound (Savannah, GA, USA) and it’s associated tributaries under permit # 29-WJH-15-147 issued by the Georgia Department of Natural Resources. Blue crabs were caught by baited commercial traps. Mud crabs were hand collected from natural oyster clusters at low tide. Blue crabs and mud crabs were housed in separate flow-through seawater systems at the Skidaway Institute of Oceanography (SkIO). Mud crabs were sorted into three size classes based on carapace width (CW): 15–20 mm, 20–25 mm, and 25–30 mm and fed an *ad libitum* diet of oyster spat every two days. Blue crabs (20–28 mm CW) were kept for at least one week prior to their use in experiments. After a brief (24–48 h) starvation period blue crabs were fed *ad libitum* quantities of mud crabs and were switched to a controlled diet two days prior to the beginning of the experiments. During this time they were fed either 5 g or 12 g whole mud crabs (20–30 mm CW) per individual every 24 h, corresponding to low and high (roughly *ad libitum*) diets, respectively. Groups of blue crabs on a given dietary regime were housed in separate tanks, and we monitored individual blue crabs during feeding to verify they consumed the food given. Oyster spat were obtained from local commercial suppliers. They were housed in flowing seawater for at least a month prior to the experiments so that they attained the proper size and were acclimated to local conditions.

### Field experiments

Field experiments were designed to examine whether blue crab diet controlled the reactive distance of mud crab prey in response to blue crab aversive chemical cues and how diet-dependent differences in cue perceptibility affected the intensity of the direct and cascading effects of aversive predator cues. These experiments employed enclosure cages to expose groups of mud crabs to blue crabs in a quasi-natural setting where we could monitor aspects of mud crab behavior and their effect on oysters.

**Figure 1:**
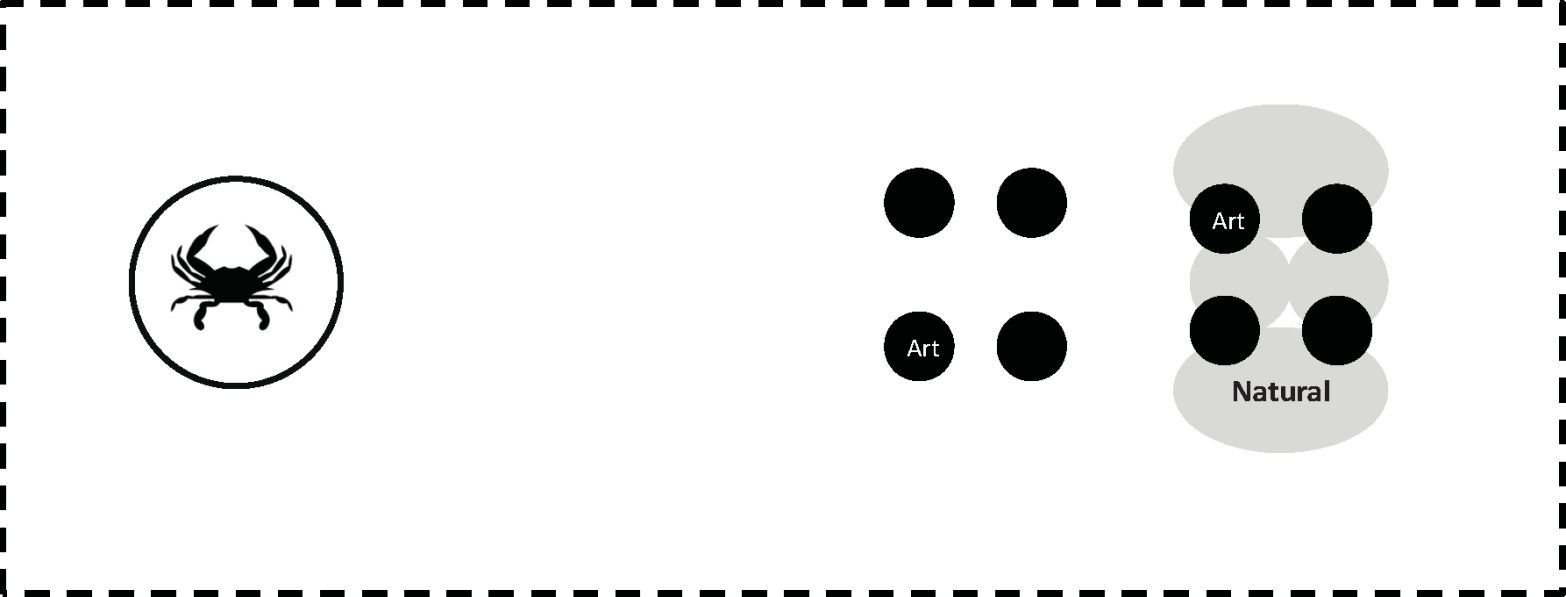
Enclosure cage. Diagram of the enclosure cage design (not to scale), showing the enclosure cage, the predator cage, the four artificial oyster clusters (Black; Art) outside and inside the natural clusters that form the refuge (Grey; Natural). Not shown is the additional predator cage located outside the enclosure, which is at the end opposite the predator cage within the enclosure. Note that only 1 of the four artificial clusters within and outside the refuge is labeled, as is only 1 of the four natural clusters forming the refuge.

Enclosures (2.2 m × 0.75 m × 0.3 m) were constructed from PVC and vexar mesh (1 cm^2^ mesh size). A small oyster reef was set up at one end of the enclosure cage to act as a refuge for mud crabs (Fig. 1). This oyster reef was a combination of four natural sun bleached oyster shell clusters (roughly 30 cm dia) interspersed with four small artificial clusters. These artificial clusters were used to control the placement of oyster spat. They were assembled by gluing together (JB Weld Marine Adhesive Putty) the external surfaces of 2 sun-bleached oyster ventral valves (10–12 cm in length) and then gluing the edges of 2 such pairs together to create a tetrad measuring approximately 10–12 cm by 5–7 cm by 2–3 cm deep. The tetrad was placed on bottom of the enclosure by pushing the umbo into the substrate so that the gape side pointed up and the internal surfaces of 4 shells faced outwards. We used marine putty to glue 4 oyster spat to the interiorsurfaces of the shells in each cluster. The reef and the embedded clusters constituted a natural refuge for mud crabs. An additional 4 artificial clusters were placed 25–35 cm away from the refuge. Each of the 8 artificial clusters contained 4 oyster spat (10–16 mm length) glued to the surface with marine epoxy. Thus, each enclosure contained 32 oyster spat (16 each within and outside of the refuge). Although mud crabs use the natural reef and the artificial clusters within them as refuge, the artificial clusters themselves generally do not present enough structure to serve in this capacity, and we rarely saw mud crabs use the isolated artificial clusters for this purpose.

In addition to the oysters, each enclosure contained mud crabs and blue crabs. A total of 15 mud crabs were used per enclosure: 8 crabs 15–20 mm CW, 4 crabs 20–25 mm CW, and 3 crabs 25–30 mm CW, which resembled the natural size distribution and density of animals in Wassaw Sound (Hill & Weissburg, 2013a). Mud crabs were marked with bright pigment to distinguish them from any ambient mud crabs that found their way into the enclosure. Each enclosure also contained a single blue crab (12–16 cm CW) fed either the low or high diet. Each blue crab was placed within its own vexar cage (0.3 m dia × 0.3 m) to prevent interaction with the mud crabs. Predator cages were set at one of five distances from the center of the oyster reef: 0.25 m, 0.5 m, 1.0 m, 1.5 m, or 2.0 m. A second caged predator with the same diet regime was placed outside the enclosure at the same distance from the reef (Fig. 1). This design balances two potentially counteracting effects of enclosures that may alter the flow; the tendency for flows to be slowed by the enclosure mesh, which reduces turbulent dispersion of chemicals, and the tendency of the mesh to produce greater mixing that increases turbulent dispersion. Chemical plumes from predator cages inside the enclosure will experience less bulk flow but not be disrupted as much by turbulence generated by the enclosure mesh compared to plumes from blue crabs in cages outside the enclosure. Acoustic Doppler flow measurements indicated that flow velocity inside the enclosures is slightly dampened by the cages, however turbulent mixing as indexed by turbulent kinetic energy (TKE) is either unaffected or slightly increased (Hill & Weissburg, 2013b). Both flow velocity and TKE are well within range of flow conditions naturally experienced at this site outside of the cages (Hill & Weissburg, 2013b; Wilson, Webster & Weissburg, 2013).

Enclosures were placed parallel to the tidal flow approximately 1 m below mean low water on a mudflat adjacent to the Priest Landing facility on the SkIO campus. The mudflat was bordered by *Spartina alternaflora* with isolated patches of aggregated oysters approximately 1–3 m in dia. Trials were deployed within a week of spring tide, when low tides were between −0.07 and −0.33 m. We have monitored flows in this area for several years, and tidal height is strongly predictive of flow speed and turbulence characteristics (Wilson, Webster & Weissburg, 2013). Flow velocity over the range of tidal amplitudes used in our study is 8–10 cm s^−1^, turbulence intensities are roughly 25%, with a turbulent kinetic energy (TKE) of around 0.65 m^2^ s^−2^. These conditions are roughly in the middle of the range for sites around Wassaw sound, except for TKE, which is on the high end.

Each trial consisted of a block of at least one replicate of every distance and diet combination, plus an additional control (no crab) treatment in which an empty predator cage was placed 0.5 m from the refuge. Enclosures were 5 m apart.

Trials lasted roughly 48 h from the time that the enclosures were placed on site and stocked with animals, which occurred during low tide. Experiments were checked after 24 h, and any blue crabs that died during this period were replaced with animals that had been fed the appropriate diet, and that had not been fed once the experiment started. A total of 18 blue crabs (out of 340) required replacement. No replicate had both crabs die in a given deployment, and no treatment had more than 3 crabs die during the entire set of field experiments. The enclosures were opened after 48 h, the mud crabs collected, and the oyster clusters placed in containers for transport back into the lab, where they were examined carefully for mud crabs and surviving oyster spat in the artificial clusters both within and outside of the refuge.

Trials occurred from May to August of 2013 and 2014. In 2014 we sometimes deployed more than one set of distances for either the high or low diet. Although this is not a completely balanced design, we were limited by the space available for the enclosures, and the time available for getting the experiment in place before the water level became too high. Sixteen enclosures (3 full distance sets plus a control) were all that were logistically and physically possible. For trials conducted in 2014, we also counted surviving oyster spat within and outside of the refuge after 24 h, in addition to counting the number of spat after 48 h as in trials conducted in 2013. The number of spat consumed after 48 h is cumulative. Since it was not necessary to remove the clusters or refuge in order to count the surviving spat, this caused minimal disturbance of the mud crabs within the enclosures, especially as mud crabs shelter well within or under the reef during low tide when counting occurred.

Aversive responses of mud crabs was evaluated by examining both the total number of oyster spat consumed and the proportion of spat consumed from the artificial clusters located outside of the refuge (# consumed outside refuge/total # consumed). Data were analyzed using a mixed model ANOVA, with distance and diet as fixed factors and date as the random (blocking) factor, using the mixed model option in JMP 11 (JMP© 11.0, SAS Institute, Cary, NC). Distance was treated as a categorical variable given that the patterns of oyster consumption with distance were non-linear, and non-linear models (logistic, exponential, etc.) provided poor fits. Because of the unbalanced design, JMP calculates error degrees of freedom using the Kenward-Roger first order approximation (JMP© 11.0, SAS Institute, Cary, NC). The proportion of spat consumed outside the refuge was arcsine transformed prior to analysis. We analyzed the 24 vs. 48 h data separately because 24 h data were collected only in 2014. Note that the control (no blue crab predator) treatments were not included in the analysis, as there is only one distance (which is effectively infinite) and adding this condition to the diet treatment group would create a non-orthogonal statistical design. Thus, for each diet we evaluated the threshold distance at which chemical cues no longer had effects by comparing the number of spat consumed and proportion of spat consumed outside the refuge at each distance to the control values using a *t*-test with a Bonferroni correction. The threshold was defined as the greatest distance at which spat consumption or proportion of spat consumed, was different from the controls.

We deployed 3 trials in 2013 and 6 in 2014. Nearly all of one trial was lost to severe weather in 2014 and was not included in the analysis. Several replicates were lost as a result of currents displacing the predator cages from their original location, and two other replicates were lost when we could not verify the artificial oyster clusters were properly identified once they returned to the lab. We could not sample some of our replicates after 24 h during one deployment in 2014 due to unusually high water levels as a result of heavy rain. We also lost one control enclosure in 2013 due to human interference but included this deployment given that oyster consumption in the no predator control enclosures exhibited low variation (see below). This left between 8 and 12 replicates for each of the various diet and distance combinations, with 5 and 7 replicates for 24 and 48 h control treatments. Data from this experiment are archived at the following site: https://smartech.gatech.edu/handle/1853/53699.

We frequently recovered the majority of the original marked crabs, with the smallest size class being the most difficult to find. Given the cages were pushed into the mud for several cm, we conclude that crabs burrowed into the mud (which is a common defense mechanism) and remained unfound even though we combed the substrate as thoroughly as time allowed. It is also possible that the animals inserted themselves so deeply into the oyster reef that we could not locate them, especially as we reused the reef clusters and were reluctant to damage them. The number of immigrants also was low; the majority of cages (>75%) lacked any unmarked mud crabs and only 2 enclosure cages had more than 2 (4 migrants were recorded twice). As noted, we rarely replaced blue crab predators after 24 h, and both blue crabs were alive after 48 h in nearly all (roughly 85%) of the enclosures. There was no pattern of either predator survival, marked crab recovery or migrant influx across treatments. Therefore we included all replicates in the analysis except as noted above.

## Results

The data from the no predator treatments indicate that mud crabs foraged effectively within the enclosure and their activity was not strongly altered by the ambient experimental conditions, collection date or other factors. After 24 h in the control treatment, mud crabs had consumed 11.6 ± 0.6 (mean ± SE) and 11.8 ± 1.02 oyster spat inside and outside the refuge, respectively, and the cumulative number of oysters spat consumed after 48 h was 13.3 ± 0.42 and 14.6 ± 0.48 inside and outside the refuge, respectively. Recall that there were 16 spat within and outside of the refuge, so the data indicate most of the oyster spat consumption occurred over the first 24 h in treatments where blue crab predators were absent. Mud crabs in the control treatment that were not subjected to chemical cues from blue crabs exploited both refuge and non-refuge locations roughly equally.

**Figure 2:**
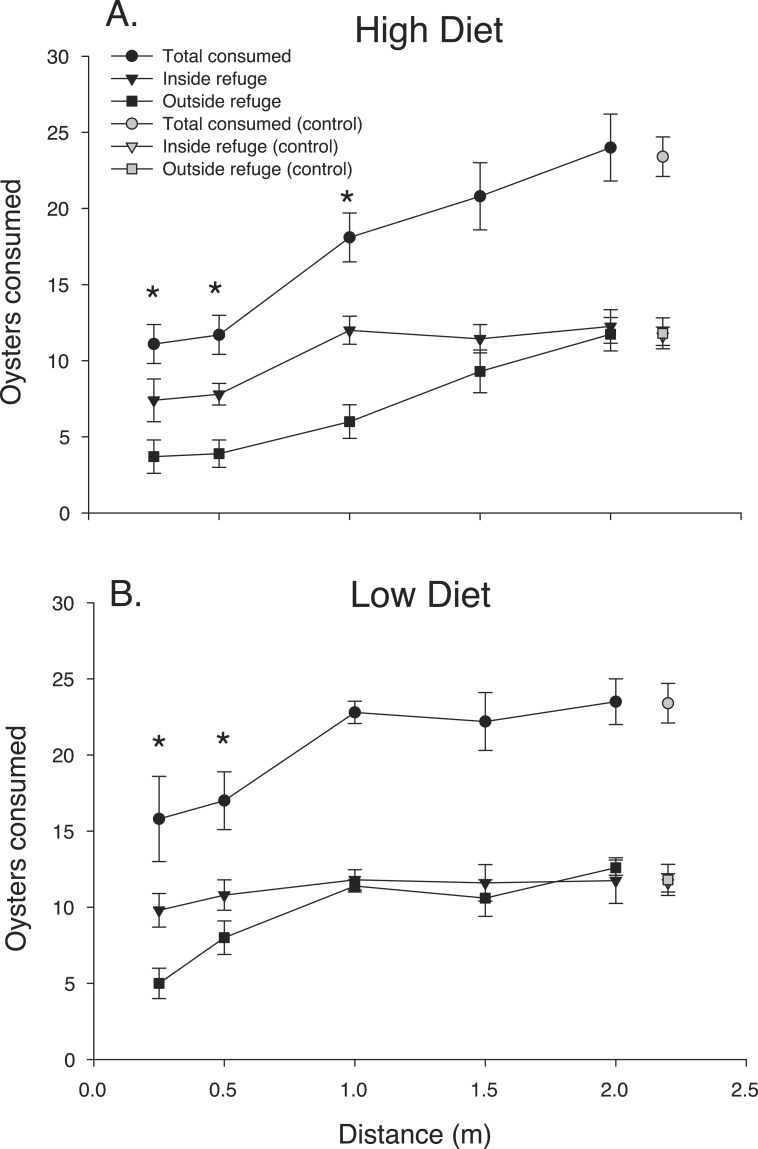
24 h consumption. Oyster consumption by mud crabs over the first 24 h as functions of distance to the predator and location, for each of the two diets. (A) High diet. (B) Low diet. Black symbols indicate total consumption (circles), and consumption inside and outside the refuge location (triangles and squares, respectively). Gray symbols indicate data from the no-predator controls. Symbols show means ±1 SE. Asterisks indicate total consumption is different from the control value using Bonferroni corrected *t*-tests. Data shown here is from trials conducted only in 2014.

Blue crab chemical cues strongly suppressed mud crab oyster consumption over the first 24 h (Fig. 2). The ANOVA revealed that distance and diet had strong effects on oyster spat consumption by mud crabs over the first 24 h interval (*F*_4,56.1_ = 18.98, *P* < 0.001; *F*_1,56.1_ = 8.09, *P* < 0.001, respectively) but there was no interaction (*F*_4,56.1_ = 0.756, *P* > 0.50). Oyster consumption increased with predator distance, and the suppressive effect of chemical cues released by blue crabs was greater in the high diet condition, particularly when distances were less than 1.0 m. The pattern of increased consumption with distance from the predator was more noticeable for the location outside the refuge, whereas consumption inside the refuge either appeared to level off at distances greater than 1.0 m (high diet) or was relatively flat overall (low diet). The threshold reactive distance was 1 m and 0.5 m for the high and low diet conditions, respectively.

**Figure 3:**
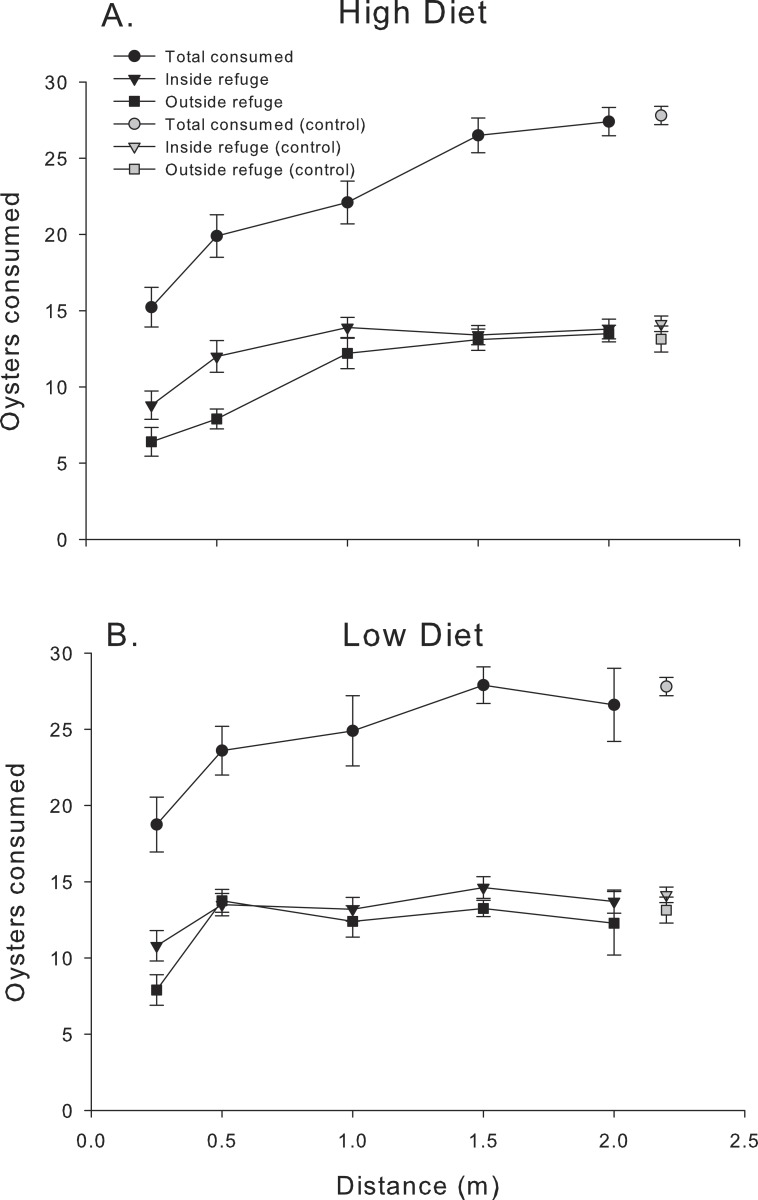
48 h consumption. Cumulative oyster consumption by mud crabs after 48 h as functions of distance to the predator and location, for each of the two diets. (A) High diet. (B) Low diet. Black symbols indicate total consumption (circles), and consumption inside and outside the refuge location (triangles and squares, respectively). Gray symbols indicate data from the no-predator controls. Symbols show means ± 1 SE. Data shown here is from trials in 2013 and 2014.

The pattern of mud crab responses changed after the first 24 h (Fig. 3). Examining the cumulative number of spat consumed after 48 h still showed an effect of distance, (*F*_4,83.2_ = 28.39, *P* < 0.001), but diet was now insignificant (*F*_1,84_ = 2.54 *P* < 0.17), despite the consistent increase in oyster consumption with distance in the high diet treatment. In contrast, low diet treatment showed much less of a distance effect. Mud crabs exposed to well-fed blue crabs seemed to show more foraging suppression compared to the low diet treatment at distances less than 1.5 m, but the effect was not apparent at the two greatest distances. There was no distance-diet interaction (*F*_4,83.1_ = 1.6, *P* < 0.20). In general, oyster consumption was much more equal at the two locations inside vs. outside the refuge than in the first 24 h.

**Figure 4:**
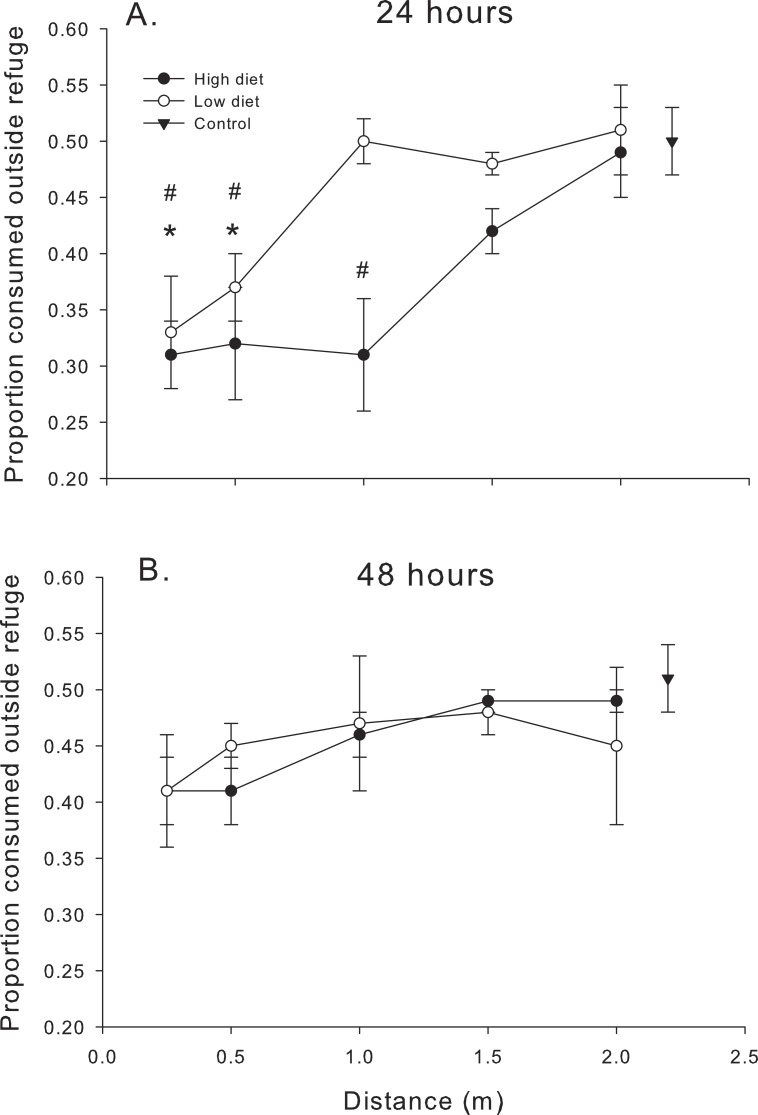
Proportion consumed outside the refuge. Proportion of oysters consumed outside the refuge (# consumed outside the refuge/total # consumed) as functions of diet and distance. (A) After 24 h. (B) After 48 h. Symbols show means ±1 SE. Asterisks indicate total consumption in low diet treatments is different from the control value using Bonferroni corrected *t*-tests. Hashmarks (#) indicate total consumption in high diet treatments is different from the control value using Bonferroni corrected *t*-tests. Data is from trials conducted in 2014 (24 h) and 2013–2014 (48 h).

The relationship between proportion of spat consumed outside the refuge (# eaten outside refuge/total # eaten) and distance confirmed some of the trends noted in Figs. 2 and 3. Proportion consumed was a function of distance (*F*_4,56.1_ = 18.99, *P* < 0.001) and diet (*F*_1,56.1_ = 8.09, *P* < 0.01) over the first 24 h (Fig. 4A). In general, mud crabs preferentially consumed spat inside the refuge when predators were <1.0 m, which is consistent with the observation that consumption inside the refuge remained relatively constant (within a diet treatment), but consumption outside the refuge increased as blue crabs were placed farther away (Fig. 2). Hence, mud crabs exploited both locations much more equally when blue crabs were far away, and the proportion consumed approached 0.5 at the 2 m distance. As noted, the effect was stronger overall in the high diet treatment. The degree of difference between the high and low diet treatments was much greater when blue crabs were less than 1 m away and diminished as the proportion consumed for both diet treatments approached 0.5 at greater distances, although there was no distance*diet interaction (*F*_4,56.1_ = 0.76, *P* > 0.50). The threshold distance at which mud crabs no longer consumed relatively more spat inside the refuge was 1.5 m and 0.5 m for the high diet and low diet, respectively.

The pattern once again changed after the first 24 h. When compared to the data over the first 24 h, oyster consumption by mud crabs inside vs. outside the refuge was more equal; the proportion consumed was closer to 0.5 for all distances and diets after 48 h (compare Figs. 4A and 4B). There was no significant effect of distance (*F*_4,83.6_ = 1.53, *P* < 0.20) or diet (*F*_1,84.4_ = 0.64, *P* < 0.40) or their interaction (*F*_4,83.5_ = 0.75, *P* < 0.40) on the proportion of oysters consumed after 48 h.

## Discussion

These experiments show clearly that the amount of prey flesh recently consumed by a predator affects response intensity and the range over which aversive responses occur; compared to predators that have consumed less prey, blue crabs consuming more prey produce an elevated response that occurs at greater distances from the predator. The results shown here are consistent with other reports showing that greater predator biomass enhances chemically mediated prey aversive responses (Relyea, 2003; Ferrari, Messier & Chivers, 2006; Hill & Weissburg, 2013b). The diet-dependent effects documented here are beneficial by allowing prey to decrease their risk more in response to predators that have dined more extensively on mud crabs and therefore allow mud crabs to calibrate their aversive behavior to the degree of potential danger. However, the response pattern also reflects some fundamental constraints on information obtainable by chemical cues. Although hungry predators might well be very threatening, they may not release dietary metabolites that are detectable (e.g., Smee & Weissburg, 2006) and thus damage-related chemical cues such as those produced by injured prey become the primary means by which threat is assessed in these cases.

Mud crab prey decreased their consumption of oysters over distances of up to 1–1.5 m when predators were well fed, and considerably less so when predators were fed less, which shows that the reactive distance of prey and the strength of the response at a particular distance is contingent on the amount of prey tissue that predators consume. The different reactive distances, as well as differences in prey response at a given distance, likely are due to greater concentrations of bioactive metabolites in predators that have consumed more prey. In fact, urine harvested directly from blue crabs that have dined on mud crabs is a potent aversive cue, and urine from blue crabs fed differing amounts of mud crab flesh differ quantitatively in their chemical signature (R Poulin, pers. comm., 2014).

Both the reactive distance to predators and the effect strength declined over time as predators metabolized their meal. Some of the reduction in response intensity after 48 h may be traceable to the fact that we did not renew the oyster spat after 24 h. Mud crabs in treatments where blue crabs were placed at distances 1 m or greater (and in the controls) consumed a substantial fraction of the available prey during this period, making oysters less available and placing an upper limit on consumption in these conditions. However, mud crab oyster consumption dramatically increased between 24-48 h outside the refuge and at distances 1 m or less from the predator in both diet treatments (compare Figs. 2 and 3). These increases represent a substantial fraction of the daily consumption in the absence of chemical cues and are large relative to the consumption over the first 24 h. Here, the decline in effectiveness of predator scent over the second 24 h interval is not likely to be the result of resource depletion since over half the spat were still unconsumed at the beginning of this interval. Thus, some part of the decreased effect of blue crab chemical cues through time likely is the result of exhausting the supply of bioactive molecules as blue crabs metabolize their meal, and this conclusion is consistent with the fact that the amount of prey consumed by blue crabs (i.e., diet) exerts strong effects. It is also possible that mud crabs habituated to the presence of the chemical cue. However, we note that blue crabs do not produce urine continuously (M Weissburg, pers. obs., 2013) and mud crabs will not be exposed to any predator scent during low tide, potentially reducing the effects of habituation. The contributions of sensory adaptation/habituation vs. depletion of bioactive metabolites remains to be determined.

Mud crabs preferentially forage for oysters within refuge habitats in the presence of chemical cues, and the ability of predator (chemical) cues to drive behaviorally mediated trophic cascades (NCEs) into refuge habitats has been documented previously (Grabowski, 2004; Grabowski & Kimbro, 2005). However, the present experiments show this effect to be contingent on the strength of the chemical cue as determined by predator diet and distance; high diets and close distances produced the strongest disparity between consumption within vs. outside the refuge, most likely because competition among mud crabs produces a cost of relying solely on resources within the refuge. Understanding the distribution of effects across environments therefore requires analysis of not simply habitat complexity, but predator density, location and cue intensity and persistence.

**Figure 5:**
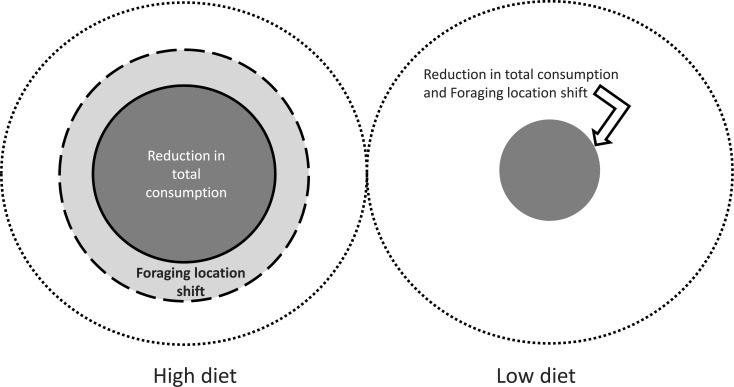
Predator sphere of influence for the effects of blue crab chemical cues. The outer most circle represents 2 m from predator, with the inner circles representing the distance over which suppressive effects are expressed over the first 24 h of the experiment. The diameter of each circle is to scale, thus the size of the circle corresponding to reduction in consumption for the high diet (1.0 m; Fig. 2A) is one half of the diameter of the largest circle that represents the 2 m limit. Threshold distances for the effects are estimated by examining the distance at which total consumption and proportion of consumption outside the refuge (Figs. 2 and 4, respectively) differs from control values for that same time period.

The role of predator diet in modulating the strength of prey responses to chemical cues, and their range, creates a complex spatial pattern of prey responses, both in terms of type and magnitude. Figure 5 illustrates the threshold distance from the predator at which particular effects occur (Figs. 2 and 4) during the first 24 h, for reduction in total consumption and preferential foraging within the refuge. Such reactive distances have also been called the predator “sphere of influence” (Turner & Montgomery, 2003) although for substrate bound animals in flow, dispersion in the vertical direction is small so the predator effect is accurately approximated as an area.

Chemical signals in flow propagate along stream parallel to flow direction, and are spread across stream by turbulent dispersion. Rahman and colleagues (2005) indicate that in flow conditions similar to our field sites, the across stream growth of the plume is proportional to *x*^0.75^, where *x* is the downstream distance. Thus, if the downstream limit of the effect is 1.5 m, we expect the plume width to expand to roughly 15 cm, based on an initial source size of 10 cm (e.g., the carapace diameter of a large blue crab) and assuming that the odor expands immediately in the wake of the crab to this size. Taking the plume width as the arc subtended by the plume, the coverage downstream represents roughly 2.5% of area of a 1.5 m circle around the predator. Even under this series of optimistic assumptions, it seems that chemical cues may be limited in their ability to affect a large number of prey in many aquatic environments with even moderate levels of water flow. Turner & Montgomery (2003) found that metabolites released by pumpkinseed sunfish (*Lepomis gibbosus*) affect the snail *Physa acuta* at distances up to roughly 3 m in a large lake, which is the only comparison we believe is available. Not unexpectedly, the threshold distance in slowly moving water where turbulent dispersion is small exceeds that observed in flow, and shows the potential importance of environmental control. Experimenters often have not paid attention to the sensory environment when examining the ecological effects of predator detection (Weissburg, Smee & Ferner, 2014). Accounting for environmental controls on the ability of prey to gather informationis required for a full understanding of the ecological impact of prey responses to risk cues.

Given that different predators will have different recent dining histories, threat perception via chemical cues can create a spatially variable landscape of differing effects and effect strengths independent of the particular flow dynamics. This landscape also will be temporally variable as predators experience different levels of success in capturing prey. The tendency for prey to aggregate near refuges can create spatial variation in NCEs in a tritrophic system, enhancing the survival of the basal prey species (Matassa & Trussell, 2011). The present results suggest that spatial variation in predator foraging success or distribution also may produce larger scale heterogeneity of NCEs independent of environmental variation affecting intermediate prey susceptibility to top level predators.

The positive relationship between blue crab predator diet and the propagating effects of mud crab behavioral changes onto oyster prey indicates an unanticipated coupling between consumptive and non-consumptive effects. Successful predation represents a CE, but only prey that are not eaten can produce behavioral or other changes that cascade to other community members, perhaps suggesting CEs and NCEs should be negatively associated; if more prey are consumed fewer are capable of responding to predator cues. This is not the case when the ability of prey to sense predators is contingent on the recent success of the predators in consuming prey; NCEs and CEs will fluctuate roughly in synchrony, albeit with some lag. Predator cue variability has strong effects because prey can rapidly alter behavior in response to changes in perceived risk (Hughes, Rooker & Kimbro, 2012). Consequently it is critical to understand how variability in response to risk cues is created. Over time, dynamic responses of intermediate prey to changes in the strength of aversive cues from predators, and the cascading effects, may be difficult or impossible to approximate by extrapolating from average or typical conditions.

We caution, however, that chemically based detection systems may be unusual in that the strength of the aversive cue is linked to the predator’s success as a consumer. Visual or auditory cues may not exhibit the same strong link between predatory success and aversive cue strength. Perhaps the ability to detect both predator foraging success and presence confers unique advantages to chemically based detection systems, since this would allow prey to accurately scale their behavior to local risk in a way not otherwise possible. We also caution that multi-modal detection of predators would reduce the degree of coupling between CE and NCE strength. Prey species can employ multi-modal detection combining visual and chemical sensing (Brown & Cowan, 2000; Chivers et al., 2001; Amo, Lopez & Martin, 2004), but the ecological effects of unimodal vs. multimodal predator detection remain poorly investigated. Certain environments may prevent multimodal detection by restricting the range of one cue relative to others, which is highly likely in the extremely turbid conditions at our field site.

## Additional Information and Declarations

### Field Study Permissions

The following information was supplied relating to field study approvals (i.e., approving body and any reference numbers): Georgia Department of Natural Resources permit #29-WJH-15-147.

### Data Availability

The following information was supplied regarding data availability: PeerJ Consumption Data: http://hdl.handle.net/1853/53699.
